# Infectious Complications in Burn Patients: A 10-Year Multicenter Study in the Petróleos Mexicanos (PEMEX) Hospital Network

**DOI:** 10.7759/cureus.103069

**Published:** 2026-02-05

**Authors:** Sandra Quintana Ponce, Erika Barlandas Quintana, Nicolas Rogelio Eric Barlandas Rendon, Mercedes Calixto Galvez, Abraham Hernán Herrera-Sánchez, Mauricio Gutierrez-Alvarez, Cuahutemoc Marquez-Espriella

**Affiliations:** 1 Faculty of Natural Sciences, Universidad Autonoma de Guerrero, Chilpancingo, MEX; 2 Department of Plastic and Reconstructive Surgery, Hospital Central Sur de Alta Especialidad de Pemex, Mexico City, MEX

**Keywords:** burns, epidemiology, infectious complications, multidrug resistance, risk factors

## Abstract

Background: Infectious complications are a leading cause of morbidity and mortality in burn patients, particularly in tertiary care centers treating complex cases. The loss of the skin barrier, systemic inflammatory response, and secondary immunosuppression increase susceptibility to colonization, sepsis, and multidrug-resistant infections. Understanding the epidemiology and timing of these complications is critical to optimize prevention and treatment strategies. However, multicenter data describing long-term infection patterns and antimicrobial resistance in burn patients from Latin American healthcare networks remain limited. Additionally, evaluating a decade-long cohort allows for a more comprehensive characterization of epidemiological trends and resistance profiles over time.

Objective: This study aims to determine the prevalence of infectious complications, microbiological profile, and associated risk factors in burn patients treated in the hospital network of Petróleos Mexicanos (PEMEX).

Methods: We conducted a multicenter, retrospective, observational study based on medical record review of burn patients hospitalized in six Petróleos Mexicanos (PEMEX) health centers across Mexico between 2014 and 2024. Demographic, clinical, and microbiological data were collected. Univariate analysis with chi-squared and Mann-Whitney U tests was performed, followed by logistic regression to identify independent risk factors associated with infection. A significance level of 5% was used.

Results: We analyzed 273 hospitalized burn patients; 71.27% were men, and the mean age was 43.6 ± 18.8 years. The trunk was the most commonly affected area (52.36%), and 21.82% had burns >20% total body surface area (TBSA). The infection rate was 13.45%, with *Pseudomonas aeruginosa* being the most frequent pathogen (7.64%), showing high resistance to tobramycin (84.21%) and imipenem (78.4%). Factors significantly associated with infection (p < 0.05) included burns >20% TBSA, intensive care unit (ICU) stay, involvement of lower extremities, and advanced age.

Conclusions: Extensive burns, ICU admission, lower extremity involvement, and older age are significant risk factors for infectious complications. The high prevalence of *Pseudomonas* and its antibiotic resistance profile highlight the need for updated antimicrobial protocols and rigorous prevention strategies in burn care.

## Introduction

Burn injuries are among the most severe forms of trauma, associated with high morbidity and mortality worldwide [1]. Infection remains the leading cause of death in hospitalized burn patients [2,3]. Thermal injury compromises the skin’s barrier function and induces systemic immune dysfunction, creating a protein-rich environment conducive to microbial colonization and sepsis [4-6]. Inhalation injury is a common concomitant finding, resulting in airway edema, increased mucus production, ulceration, and obstruction of small airways by fibrinous debris, further impairing immune defense and increasing the risk of pneumonia [7].

The risk of infection is influenced by burn extent, depth, intensive care unit (ICU) admission, mechanical ventilation, and prolonged hospitalization [3,8]. Multidrug-resistant organisms (MDROs) further complicate management, emphasizing the importance of local epidemiological data to guide preventive and therapeutic strategies [9-11].

This study aimed to determine the prevalence of infectious complications, describe the microbiological profile, and identify independent risk factors among hospitalized adult burn patients treated across the Petróleos Mexicanos (PEMEX) hospital network in a multicenter cohort.

## Materials and methods

We conducted a multicenter retrospective cohort study using systematically reviewed medical records of burn patients hospitalized between 2014 and 2024 across six Petróleos Mexicanos (PEMEX) hospitals in Mexico.

Study design and setting

We conducted a multicenter retrospective cohort study using systematically reviewed medical records of burn patients hospitalized between 2014 and 2024 across six Petróleos Mexicanos (PEMEX) hospitals in Mexico. Patients were followed throughout hospitalization to identify the occurrence of infectious complications.

Study population

We included adult patients (>18 years) admitted for burn treatment during the study period. Patients with incomplete records or follow-up shorter than six months were excluded.

Data collection

Demographic and clinical variables collected included age, sex, burn mechanism (flame, electrical, scald, chemical, and friction), total body surface area (TBSA) burned (<20% or ≥20%), inhalation injury, intensive care unit (ICU) admission, mechanical ventilation, hospital stay, work disability days, and mortality.

Infectious complications were recorded, including infection site, pathogen isolated, antimicrobial susceptibility patterns, sepsis, and need for debridement. Infectious complications were identified based on documented clinical diagnoses supported by microbiological confirmation when available, following institutional protocols aligned with internationally accepted burn care practices. Clinical specimens were collected according to standard care practices at each institution and processed using established microbiological methods.

Statistical analysis

Patients were categorized according to the presence or absence of infectious complications. Categorical variables were compared using the chi-square test or Fisher’s exact test, as appropriate, while continuous variables were analyzed using Student’s t-test or Mann-Whitney U test based on data distribution. Continuous variables were assessed for normality using the Shapiro-Wilk test and are presented as mean ± standard deviation (SD) or median with interquartile range, as appropriate.

Cases with incomplete key variables were excluded from regression analyses. Missing data were minimal (<5%) and were handled using complete-case analysis.

Univariate analyses were initially performed to identify variables associated with infection. Variables with a p-value < 0.05, as well as those considered clinically relevant, were entered into a multivariable logistic regression model to identify factors independently associated with infectious complications. Clinically relevant variables were evaluated as potential confounders during model construction, and multicollinearity was assessed prior to model development. To minimize the risk of overfitting, the number of predictors included in the model was restricted according to the number of outcome events. Given the number of infection events observed, the number of predictors included in the final model was intentionally limited to preserve model stability and reduce the risk of overfitting.

Model fit was assessed using the Hosmer-Lemeshow goodness-of-fit test. Adjusted odds ratios (OR) with 95% confidence intervals (CI) were reported. All tests were two-tailed, and a p-value < 0.05 was considered statistically significant. Statistical assumptions were verified prior to analysis. All analyses were performed using IBM SPSS Statistics version 25.0 (IBM Corp., Armonk, NY).

Ethical considerations

This study was approved by the institutional ethics committee and conducted in accordance with the Declaration of Helsinki and Mexican regulations. All medical records were fully de-identified prior to data extraction, ensuring that no personal identifiers were included.

## Results

A total of 273 burn patients were included. Of these, 196 (71.27%) were men and 79 (28.73%) women, with a mean age of 43.6 ± 18.8 years. The median hospital stay was 14.5 days.

The trunk was the most affected region (52.36%), followed by upper extremities (32.73%), head and neck (13.82%), and lower extremities (10.91%). Most burns were second-degree (68.73%), followed by third-degree (28%) and first-degree (2.55%). Burns >20% TBSA were seen in 21.82% of patients (Table 1). Patient care frequency by hospital is shown in Table 2.

**Table 1:** Demographic and Clinical Characteristics of the Study Population SD: standard deviation, TBSA: total body surface area

Variable	Descriptor	Value
Total patients	Number	273
Sex (men)	Number (%)	196 (71.27%)
Sex (women)	Number (%)	79 (28.73%)
Mean age (years)	Mean ± SD	43.6 ± 18.8
Median hospital stay (days)	Median	14.5
Burn region (trunk)	Number (%)	144 (52.36%)
Burn region (upper extremities)	Number (%)	90 (32.73%)
Burn region (head and neck)	Number (%)	38 (13.82%)
Burn region (lower extremities)	Number (%)	30 (10.91%)
Burn depth (first degree)	Number (%)	7 (2.55%)
Burn depth (second degree)	Number (%)	189 (68.73%)
Burn depth (third degree)	Number (%)	77 (28%)
Burns >20% TBSA	Number (%)	60 (21.82%)
Infection rate	Number (%)	37 (13.45%)

**Table 2:** Patient Care Frequency by Hospital

Hospital	Frequency	Percentage
Hospital Central Norte, Mexico City	16	5.82%
Hospital Central Sur, Mexico City	115	41.82%
Tabasco	39	14.18%
Poza Rica	29	10.55%
Guanajuato	21	7.64%
Tamaulipas	13	4.73%
Minatitlán	36	13.09%

The overall infection rate was 16.1% (44/273). The most frequent pathogen was *Pseudomonas aeruginosa* (7.64%), followed by *Staphylococcus haemolyticus*, *Staphylococcus epidermidis*, *Enterococcus faecalis*, and *Staphylococcus hominis* (Table 3). High resistance rates were observed for tobramycin (84.21%), ciprofloxacin (59.46%), vancomycin (35.14%), clindamycin (81.58%), and imipenem (78.4%) (Table 4).

**Table 3:** Pathogens Isolated and Resistance Rates

Pathogen	Frequency	Percentage (%)
Pseudomonas aeruginosa	21	7.64
Staphylococcus haemolyticus	5	1.82
Staphylococcus epidermidis	5	1.82
Enterococcus faecalis	4	1.45
Staphylococcus hominis	2	0.73

**Table 4:** Antibiotic Resistance Rates

Antibiotic	Resistance frequency	Resistance (%)
Tobramycin	16	84.21
Ciprofloxacin	22	59.46
Vancomycin	13	35.14
Clindamycin	7	81.58
Imipenem	9	78.40

Univariate analysis identified several variables associated with infectious complications (Table 5). Subsequently, multivariable logistic regression was performed to adjust for potential confounders, including clinically relevant variables. Burns involving >20% total body surface area (TBSA), intensive care unit admission, lower extremity burns, and advanced age remained independently associated with infection after adjustment.

**Table 5:** Risk Factors for Wound Infection in the Study Population TBSA: total body surface area, ICU: intensive care unit, CI: confidence interval

Variable	Not infected (number (%))	Infected (number (%))	Chi-square	p-value	Odds ratio	95% CI
Total patients	229 (83.88%)	44 (16.12%)	-	-	-	-
>60 years old	30 (13.10%)	17 (38.63%)	16.88	<0.001	4.18	2.04-8.56
Diabetes mellitus	65 (28.38%)	8 (18.18%)	1.96	0.160	0.56	0.24-1.27
Arterial hypertension	61 (26.63%)	10 (22.72%)	0.293	0.588	0.81	0.34-1.73
Burns >20% TBSA	45 (19.65%)	15 (34.09%)	4.48	0.034	2.11	1.04-4.27
Burn in the head and neck	14 (6.11%)	4 (9.09%)	1.02	0.312	0.57	0.19-1.70
Burn in the trunk	120 (52.40%)	24 (54.54%)	0.068	0.790	1.09	0.57-2.08
Burn in the upper extremities	74 (32.31%)	16 (36.36%)	0.274	0.601	1.19	0.61-2.34
Burn in the lower extremities	18 (7.86%)	12 (27.27%)	14.22	0.000	4.39	1.93-9.97
ICU admission	11 (4.80%)	12 (27.27%)	24.15	0.000	7.43	3.02-18.24
Burn depth (first degree)	7 (3.05%)	0 (0%)	-	-	-	-
Burn depth (second degree)	162 (70.74%)	27 (61.36%)	-	-	-	-
Burn depth (third degree)	60 (26.20%)	17 (38.63%)	3.83	0.147	1.53	0.11-1.23

## Discussion

This study provides a comprehensive analysis of infectious complications among burn patients in a multicenter Mexican cohort over a 10-year period. Our findings reaffirm that infection remains a major cause of morbidity in burn units, with a prevalence of 13.45%, aligning with rates reported in similar resource-limited settings [2,4].

The identification of burns involving >20% total body surface area (TBSA), intensive care unit (ICU) admission, lower extremity involvement, and advanced age as independent risk factors for infection is consistent with previous studies [5-7]. Brusselaers et al. demonstrated that extensive burns (>20%-40% TBSA) were associated with up to a 10-fold increase in infection risk, reflecting the profound immunosuppression and tissue damage present in such patients [1]. Similarly, our results emphasize the relevance of burn size as a reliable predictor of infectious morbidity.

We also observed a significant association between lower extremity burns and infection, which has been previously noted in smaller series [9]. This may be attributed to the anatomical challenges of maintaining asepsis in the lower limbs, greater exposure to environmental contaminants, and impaired perfusion that hinders wound healing. Future studies could further explore the role of regional blood flow and tissue oxygenation in infection risk stratification.

ICU admission and invasive support measures such as mechanical ventilation and central venous catheters are well-recognized contributors to nosocomial infections in critically ill burn patients [3,8]. Our data support this observation, underlining the need for stringent infection control policies in intensive care environments, particularly in patients with high TBSA burns and prolonged stays.

Advanced age was significantly associated with infectious complications, consistent with findings by Alp et al. [8] and others. Aging has been linked to diminished immune competence, delayed wound healing, and increased comorbid burden, which may contribute to a higher susceptibility to infection. Given the observational design of this study, these findings should be interpreted as associations rather than causal relationships. These results may help inform risk stratification and support closer monitoring of elderly burn patients.

Of particular concern was the predominance of *Pseudomonas aeruginosa*, accounting for more than half of the isolated pathogens, with alarmingly high resistance rates to commonly used antibiotics such as tobramycin (84.21%), imipenem (78.4%), and ciprofloxacin (59.46%). These findings echo global concerns over multidrug-resistant *Pseudomonas* in burn care, as highlighted by the World Health Organization (WHO) priority pathogen list [10]. The high resistance rates observed in our cohort may reflect both the selective pressure of empirical broad-spectrum antibiotic use and inadequate infection control practices. Implementation of antimicrobial stewardship programs and periodic local antibiograms are critical to guide rational therapy, prevent further resistance, and improve patient outcomes [11].

Beyond individual patient management, our findings carry institutional and public health implications. The identification of specific risk factors enables the development of targeted surveillance and prophylactic interventions, for example, enhanced barrier nursing care and early wound excision in patients with large TBSA burns, or tailored antimicrobial regimens in units with high multidrug-resistant organism (MDRO) prevalence.

This study is subject to several limitations inherent to its retrospective design, including potential underreporting of infections and the inability to establish causality. Furthermore, variations in microbiological surveillance practices among participating centers may have influenced pathogen detection rates. Additionally, this study was conducted within the Petróleos Mexicanos (PEMEX) hospital network, which follows specific care protocols and serves a defined patient population; therefore, the findings may not be fully generalizable to other healthcare settings and should be interpreted in the context of similar tertiary burn centers. Future prospective multicenter studies with standardized definitions and longitudinal follow-up are warranted to validate our findings and quantify the impact of preventive measures. Additionally, excluding patients with incomplete records or limited follow-up may have introduced selection bias. Furthermore, residual confounding from unmeasured variables such as nutritional status, timing of surgical interventions, or duration of invasive device use cannot be entirely ruled out.

Our results underscore the ongoing need for improved infection control protocols, timely wound management, and updated antimicrobial guidelines informed by local resistance patterns. Further research should explore the efficacy of novel therapies, such as topical antimicrobials and adjunctive immunomodulators, particularly in elderly and high-TBSA burn patients. Although multivariable analysis was performed, these findings should be interpreted as associations rather than definitive causal relationships.

## Conclusions

Extensive burns (>20% total body surface area), intensive care unit admission, lower extremity involvement, and advanced age were significantly associated with infectious complications in hospitalized burn patients. The high prevalence of multidrug-resistant *Pseudomonas aeruginosa* underscores the need for ongoing microbiological surveillance and antimicrobial stewardship. Given the observational nature of this study, these findings should be interpreted as associations rather than causal relationships and may help inform risk stratification and preventive strategies in similar burn care settings.
